# Misunderstandings and misinterpretations: Antimicrobial use and resistance in salmon aquaculture

**DOI:** 10.1111/1758-2229.13147

**Published:** 2023-03-19

**Authors:** Felipe C. Cabello, Ana R. Millanao, Ivonne Lozano‐Muñoz, Henry P. Godfrey

**Affiliations:** ^1^ Department of Pathology, Microbiology and Immunology New York Medical College Valhalla New York USA; ^2^ Instituto de Farmacia, Facultad de Ciencias Universidad Austral de Chile Valdivia Chile; ^3^ Departamento de Producción Animal, Facultad de Ciencias Agronómicas Universidad de Chile Santiago Chile; ^4^ Department of Pathology (retired) New York Medical College Valhalla New York USA

## Abstract

The exponential growth of aquaculture over the past 30 years has been accompanied by a parallel increase in the use of antimicrobials. This widespread use has had negative effects on animal, human and environmental health and affected the biodiversity of the environments where aquaculture takes place. Results showing these harmful effects have been resisted and made light of by the aquaculture industry and their scientific supporters through introduction of misunderstandings and misinterpretations of concepts developed in the evolution, genetics, and molecular epidemiology of antimicrobial resistance. We focus on a few of the most obvious scientific shortcomings and biases of two recent attempts to minimise the negative impacts of excessive antimicrobial use in Chilean salmon aquaculture on human and piscine health and on the environment. Such open debate is critical to timely implementation of effective regulation of antimicrobial usage in salmon aquaculture in Chile, if the negative local and worldwide impacts of this usage are to be avoided.

## BACKGROUND

The use of antimicrobials as growth promoters, prophylactics and therapeutics in terrestrial animal husbandry has been repeatedly shown to result in capture of mobile genetic elements and antimicrobial resistance genes derived from agricultural environments by human commensal and pathogen resistomes (Anderson, 1968; Datta, 1965; Davis et al., 2011; Larsson & Flach, 2022; Marshall & Levy, 2011; Smith, 1974). Such antimicrobial use has been documented to have an appreciable negative impact on human, animal and environmental health as a result of the loss of antimicrobial efficacy in all their uses (Innes et al., 2020; Marshall & Levy, 2011; Robinson et al., 2016; Silbergeld et al., 2008). In patients, this loss has been associated with complications in the treatment of infectious disease and increased mortality (Innes et al., 2020; Marshall & Levy, 2011; Robinson et al., 2016).

Antimicrobials are also used in aquaculture. Their annual use in this activity worldwide currently accounts for approximately 5.7% of all antimicrobials used in animal husbandry, veterinary medicine, and human medicine. This usage has been predicted to increase by a third between 2017 and 2030 (Schar et al., 2020). Worldwide, antimicrobials are considerably more intensively used in aquaculture in terms of kg of treated biomass (165 mg kg^−1^) than in terrestrial food animal production (140 mg kg^−1^) or patient care (91.7 mg kg^−1^) (Schar et al., 2020; Tiseo et al., 2020). Not surprisingly, this heavy usage has been associated with negative effects similar to those arising in terrestrial farming (Cabello et al., 2013, 2016; Heuer et al., 2009).

A recent example is the emergence and global dissemination of plasmid‐mediated *mcr* colistin resistance from selection by antimicrobials used in aquacultural activities in China (Cabello & Godfrey, 2018; Manageiro et al., 2022; Shen et al., 2020). Other examples include the emergence of clinically relevant, plasmid‐mediated quinolone resistance genes *qnrA* and *qnrS* apparently originating in aquatic *Shewanella* and *Vibrio* (Jacoby et al., 2014; Rodríguez‐Martínez et al., 2016). Such spread is mediated by a wide range of mobile genetic elements and naked DNA selected by aquacultural activities (Baquero et al., 2021; Cabello et al., 2013, 2016). These examples underscore the potential ability of bacteria of the aquatic environment and their mobile genetic elements to share antimicrobial resistance genes and genetic determinants with terrestrial animal and human commensals and pathogens (Cabello et al., 2013, 2016; Miller & Harbottle, 2018; Vincent et al., 2021). The link between aquaculture and the human resistome is further underscored by the many reports of contamination of aquacultural products raised for human consumption with such antimicrobial‐resistant commensal and pathogenic bacteria (Chen et al., 2021; Feng et al., 2012; Helsens et al., 2020; Kumar et al., 2016; Noor Uddin et al., 2013; Radu et al., 2003; Ryu et al., 2012).

## MISUNDERSTANDINGS AND MISINTERPRETATIONS

The recent papers of Avendaño‐Herrera et al. (2022) and Salgado‐Caxito et al. (2022) are noteworthy in minimising and underestimating the harmful impacts of excessive antimicrobial use in aquaculture in general and in salmon aquaculture in Chile in particular. This response recalls those of critics of the initial and subsequent studies of antimicrobial use in terrestrial farming (Anderson, 1968; Datta, 1965; Davis et al., 2011; Innes et al., 2020; Marshall & Levy, 2011; Smith, 1974). Despite the universality of the molecular genetics and evolutionary tenets underlying the development of antimicrobial resistance after antimicrobial use, these publications challenge the applicability of this extensive epidemiological and microbiological evidence for the negative impacts of excessive antimicrobial use to aquaculture. This challenge overlooks repeated demonstrations of sharing of the components of the genetic mobilome and antimicrobial resistance genes between bacteria of the aquatic environment and commensal and pathogenic bacteria of terrestrial animals and humans (Buschmann et al., 2012; Cabello, 2006; Cabello et al., 2013, 2016; Cantas et al., 2013; Lamy et al., 2022; Shah et al., 2014; Shen et al., 2020; Sørum, 2006; Sørum & L'Abée‐Lund, 2002; Tomova et al., 2015; Vincent et al., 2021).

## ANTIMICROBIAL USE IN SALMON FARMING IN CHILE

Avendaño‐Herrera et al. (2022) state there is a clear‐cut tendency of decreased antimicrobial use in Chilean salmon aquaculture between 2016 and 2021. This does not appear to be the case. While the amounts used varied from year to year during this time period, salmon aquaculture in Chile used 22% more tons of antimicrobials in 2021 than in 2020 (463 vs. 380 tons) (Servicio Nacional de Pesca [Sernapesca], 2021, 2022). Interestingly, this *increase* in antimicrobial usage was accompanied by an 8% *decrease* in salmon production in 2021. Antimicrobial usage per kilogram of salmon produced in Chile in 2021, 469 mg kg^−1^ (Sernapesca, 2021, 2022), was nearly three times its average usage in all aquaculture worldwide (165 mg kg^−1^) and 17.3 times its average usage in salmon aquaculture worldwide (27.0 mg kg^−1^) (Schar et al., 2020). Avendaño‐Herrera et al.'s ascribing farm effluents and agricultural manure as potential sources of antimicrobial residues and antimicrobial‐resistant bacteria and their antimicrobial resistance genes as sources contaminating salmon aquaculture in Chile is implausible: their use in salmon aquaculture is hundreds of times greater than their use in human or terrestrial veterinary medicine (Millanao et al., 2018; Sernapesca, 2021; Soto, 2022). In this connection, over 493 tons of florfenicol were imported for veterinary use in Chile in 2020, 74% of which were stated by Sernapesca to be used in aquaculture, while not quite 0.1 tons of chloramphenicol were imported for human use (Sernapesca, 2021; Soto, 2022). Fenicol use in salmon aquaculture in Chile is thus clearly the strongest selective activity for antimicrobial resistance there. Avendaño‐Herrera et al. further suggest that the governmental regulators, Servicio Agricola Ganadero (SAG) and Sernapesca, provide adequate control of antimicrobial use in salmon farming in Chile. While SAG reported sales of 344 tons of florfenicol for use in aquaculture, Sernapesca reported the use of 365 tons, 6% more, in this activity (SAG, 2020; Sernapesca, 2021; Soto, 2022). The significant difference in aquacultural florfenicol use in 2020 published by these two regulatory agencies belies this contention (SAG, 2020; Sernapesca, 2021; Soto, 2022), and indicates a need for improvement in this area.

Avendaño‐Herrera et al.'s extensive attempts to lessen the role of excessive antimicrobial use in salmon aquaculture in the evolution of antimicrobial resistance are based on questionable readings and interpretations of the vast literature of microbial evolution. For example, they state that *Piscirickettsia salmonis* is an apparently obligate intracellular bacterium, yet they also correctly point out that *P. salmonis* can be cultured in cell‐free medium. They also state that the putative intracellular location of *P. salmonis* results in heavy use of antimicrobials in aquaculture to overcome this hurdle, yet they appear not to realise that *P. salmonis* may also have a transient extracellular location in the host (much as occurs with *Salmonella*) where it may be reached by antimicrobials (Cabello, 1998; Casadevall & Fang, 2020; Silva & Pestana, 2013). Moreover, the classes of antimicrobials used to treat *P. salmonis*, tetracyclines and fenicols, readily penetrate eukaryotic cells and are successfully used to treat other facultative and obligate intracellular pathogens (Blanton, 2019; Pea, 2018; Smadel, 1963). This suggests the refractoriness of *P. salmonis* to treatment is perhaps the result of the presence of genetic and phenotypic resistance factors and/or other as yet unrecognised causes (Cabello & Godfrey, 2019; Jakob et al., 2014; Levipan et al., 2020; Price et al., 2016).

It is unclear why Avendaño‐Herrera et al. claim that *P. salmonis* is a problem in countries of the Northern Hemisphere when it is not (Cabello & Godfrey, 2019). Their subsequent claim that *P. salmonis* is not an emergent pathogen is similarly unclear as this organism is present in wild (free‐ranging) fish in Chile where it causes no disease and produces no pathology (Cabello & Godfrey, 2019; Pérez et al., 1998). Its emergence as a pathogen in salmon aquaculture seems likely related to shortcomings in husbandry such as poor smolt quality (Pincinato et al., 2021), unhealthy high densities of fish in pens and dysbiosis triggered by unrelenting heavy use of antimicrobials (Cabello & Godfrey, 2019).

Virulence is a complex emergent property that depends on many genes and factors, properties of the host and situations in the environment (Casadevall et al., 2011; Casadevall & Pirofski, 2009; Le Gall et al., 2007). In their incomplete review of the literature on virulence and pathogenesis, Avendaño‐Herrera et al. appear to confuse the harbouring of a potential virulence gene by a bacterial cell with the virulence of this cell. If all bacteria displaying one, two or more potential virulence properties were considered pathogens, a large proportion of bacterial commensals would be misleadingly considered pathogens requiring treatment. Their claim that antimicrobial‐resistant bacteria are not generated de novo overlooks current knowledge of evolution of antimicrobial resistance and its genetic platforms as well as the fact that following the industrial production of antimicrobials and their extended use in animals and humans, capture of antimicrobial resistance genes and mobile genetic elements of the mobilome from the environmental resistome resulted in emergence of novel antimicrobial‐resistant bacteria and antimicrobial resistance genes (Baquero et al., 2021; Davies & Davies, 2010; Hughes & Datta, 1983; MacLean & San Millan, 2019; Perry et al., 2016). Antimicrobial resistance genes may also be potentially generated de novo from yet uncharacterised DNA sequences (Knopp et al., 2019). Most bacterial pathogens isolated from humans before industrial antimicrobial usage became widespread lacked an abundance of plasmids and the multiple variability of resistances generated in response to this use (Falkow, 1975; Hughes & Datta, 1983). While antimicrobial resistance genes and mobile genetic elements may have a fitness cost, this cost disappears in the presence of antimicrobials (Davies & Davies, 2010). Many of these genetic combinations do not in fact have a fitness cost and they can be passed on to their bacterial descendants by vertical and by horizontal gene transfer, thus facilitating their perpetuation in bacterial communities (Davies & Davies, 2010; MacLean & San Millan, 2019).

Avendaño‐Herrera et al. attempt to counter the extensive evidence for aquaculture as being a source of antimicrobial‐resistant bacteria and antimicrobial resistance genes for humans and animals by claiming there is insufficient knowledge to ascertain the validity of this evidence. Even were this the case, it would not invalidate the existence of such processes (Buschmann et al., 2012; Cabello, 2006; Cabello et al., 2013, 2016; Cantas et al., 2013; Shah et al., 2014; Sørum, 2006; Tomova et al., 2015; Topp et al., 2018). They also overlook evidence for the presence of huge amounts of antimicrobial‐resistant bacteria in the intestines of antimicrobial‐treated salmon and other fish as well as in the marine sediments in the areas where these antimicrobials are used (Buschmann et al., 2012; Higuera‐Llantén et al., 2018) and evidence that aquaculture may be a source of antimicrobial‐resistant zoonotic pathogens (Cabello et al., 2013; Weir et al., 2012; Ziarati et al., 2022). In glossing over the problems of resistance and the potential for selection and ecotoxicity by antimicrobials to other living beings of the aquatic environment, some consumed by humans (Fortt et al., 2007; Kovalakova et al., 2020; Kumar et al., 2019; Pavón et al., 2022), Avendaño‐Herrera et al. hope to provide support for the expansion of their use in this activity.

## ASSESSMENT OF RISK OF ANTIMICROBIAL RESISTANCE IN HUMAN CONSUMERS OF SALMON RAISED USING LARGE AMOUNTS OF ANTIMICROBIALS

In contrast to the expansive review of the effects of antimicrobial usage in Chilean salmon farming provided by Avendaño‐Herrera et al. (2022), Salgado‐Caxito et al. (2022) focus on assessing antimicrobial resistance in human consumers of fillets from these Chilean salmon. In the absence of excessive antimicrobial use in farming of terrestrial and aquatic animals, selection for antimicrobial‐resistant organisms would be minimal and the well‐established impacts of antimicrobial use in selecting for antimicrobial resistance would make such a study unnecessary (Davis et al., 2011; Marshall & Levy, 2011; Millanao et al., 2018). Because antimicrobial usage in salmon aquaculture in Chile remains high and continues to increase, Salgado‐Caxito et al.'s analysis of the effects of this increase on antimicrobial resistance in humans is highly appropriate. Unfortunately, a risk assessment study based only on animal health expert opinions has multiple limitations.

In describing the impact of antimicrobial use in terrestrial food animals on selection of resistance, Salgado‐Caxito et al. do not mention that the reduction of antimicrobial use in Denmark and the Netherlands has been accompanied by a *decrease* in isolation of antimicrobial‐resistant bacteria in animals and humans (Aarestrup, 2015; Agersø & Aarestrup, 2013; Andersen et al., 2020). This provides an additional reason for attempting to clearly relate antimicrobial use in aquaculture to antimicrobial resistance relevant to humans. At the same time, it also critically undermines their statement as to whether reduction in antimicrobials in food animal production could lower the risk of humans acquiring antimicrobial‐resistant bacteria from eating animal products raised using antimicrobials.

It is important to note that antimicrobials not only select for resistant bacterial pathogens, but they also select for resistant commensals. Any such commensals potentially contaminating salmon meat will be ingested by humans and may become a component of their normal intestinal flora (Juricova et al., 2021; Li et al., 2022; McInnes et al., 2020). These antimicrobial‐resistant commensals, some potentially with new and undescribed antimicrobial resistance genes from the aquatic environment, will also have the ability to transmit their resistances to human pathogens in the gut, either spontaneously or during treatment of infections, because antimicrobials will stimulate horizontal gene transfer (Cabello et al., 2016; Heuer et al., 2009; Juricova et al., 2021; McInnes et al., 2020). This risk factor is missing in Salgado‐Caxito et al.'s analysis and is one that should have been included to fully appreciate the risks of antimicrobial use in salmon aquaculture.

Only 1.3% of the over 463 tons of antimicrobials used in salmon aquaculture in Chile in 2021 were used in freshwater aquaculture (Sernapesca, 2022). This strongly indicates the major risk regarding the selection of antibiotic‐resistant bacteria and antimicrobial resistance genes lies in the saltwater aquaculture phase (Avendaño‐Herrera et al., 2022; Cabello et al., 2020; Salgado‐Caxito et al., 2022). Moreover, antimicrobials used in the oceanic phase of salmon aquaculture are more likely to select for antimicrobial resistances closer to the consumer, since salmon fillets are prepared from salmon grown in saltwater and not in freshwater. It is also well known that fish produced after the use of antimicrobials have a bacterial flora with high numbers of multiple antimicrobial‐resistant bacteria (He et al., 2022; Higuera‐Llantén et al., 2018; Navarrete et al., 2008; Sáenz et al., 2019).

Antimicrobial treatments have been repeatedly shown to be *ineffective* in controlling *P. salmonis* infections in cultured salmon (Avendaño‐Herrera et al., 2022; Cabello & Godfrey, 2019; Jakob et al., 2014; Price et al., 2016). Despite Salgado‐Caxito et al.'s claim that ‘data obtained until 2020 have shown no resistant isolates of *P. salmonis* to oxytetracycline or florfenicol’, such resistance has in fact been demonstrated by changes in MIC to these agents (Contreras‐Lynch et al., 2017; Henríquez et al., 2016). In addition, a transferable plasmid isolated from an oxytetracycline‐resistant strain of *P. salmonis* (p3PS10) contained functional genes for resistance to chloramphenicol, tetracyclines, aminoglycosides and sulfonamides (Bohle et al., 2017; Saavedra et al., 2018) and chromosomally encoded resistance to quinolone has been observed (Henríquez et al., 2015). By concentrating their risk assessment solely on bacteria potentially resistant to oxytetracycline and florfenicol, Salgado‐Caxito et al. overlook the fact that molecular arrangements of multiple antimicrobial resistance genes in the mobilome would insure selection of multiple antimicrobial resistances besides oxytetracycline and florfenicol (Cabello et al., 2013; Marshall & Levy, 2011). Because *P. salmonis* is not known to be a human foodborne pathogen, their analysis confuses piscine health with human food safety as represented by antimicrobial residues and antimicrobial‐resistant bacteria in salmon tissues (Heredia & García, 2018).

A number of other points in Salgado‐Caxito et al.'s text and references are problematic. Chile annually produces close to 1,000,000 rather than 1000 tons of salmon, none of the references cited as mentioning the presence of antimicrobial‐resistant bacteria in treated salmon fillets do so, and contrary to what the text indicates, none of the cited references directly delve into the anthropogenic sources (including aquaculture) of antimicrobials in the environment. It is also not clear whether Salgado‐Caxito et al.'s risk assessment included any of the many publications documenting the presence of antimicrobial‐resistant commensals and potential pathogens or the presence of antimicrobial residues in fillets from fish raised with antimicrobials (Chen et al., 2021; Doğan et al., 2020; Feng et al., 2012; Griboff et al., 2020; Helsens et al., 2020; Kumar et al., 2016; Noor Uddin et al., 2013; Radu et al., 2003; Ryu et al., 2012). Such information is essential to their risk analysis and should have been included.

While the risk to human health of consumption of salmon fillets from fish raised using excessive antimicrobials may be low, some of the above points cast doubt on the certainty of Salgado‐Caxito et al.'s conclusions. One approach to correcting these shortcomings would involve quantitating antimicrobial‐resistant bacteria and antimicrobial resistance genes in commercially available salmon fillets and then comparing DNA sequences of the resistome and mobilome of these bacteria with those of the total aquatic environment (including fish) and those of commensals and pathogens of the human intestinal microbiome in people who consumed such fillets (Aguiar‐Pulido et al., 2016; Bengtsson‐Palme et al., 2017; Tümmler, 2020; Waskito et al., 2022). Neither Avendaño‐Herrera et al., nor Salgado‐Caxito et al., discuss the food safety risk that this antimicrobial use generates by contaminating other aquacultural products such as mussels and wild fish with antimicrobial residues and antimicrobial‐resistant bacteria (Fortt et al., 2007; Pavón et al., 2022; Ramírez et al., 2022).

## CONCLUSIONS

We agree with Avendaño‐Herrera et al. and Salgado‐Caxito et al. that more aggressive measures need to be implemented to reduce excessive antimicrobial use in salmon aquaculture. However, such measures need to be within the framework of accurate and well‐accepted scientific knowledge provided by evolutionary theory, microbial genetics, the One Health paradigm and the precautionary principle. While Avendaño‐Herrera et al. and Salgado‐Caxito et al. are not the first to attempt to misquote and minimise the large body of published scientific work that runs counter to their opinions and agenda (Alday et al., 2006; Finland, 1975; Hearings Before the Subcommittee on Investigations and Oversight of the Committee on Science and Technology, 1984; Phillips et al., 2004), none of these efforts have had any influence in modifying well‐established scientific concepts in this area (Brunton et al., 2019; Cabello et al., 2016; Chiller et al., 2004; Heuer et al., 2009; Jensen et al., 2004; Karp & Engberg, 2004; Lozano‐Muñoz et al., 2021; Price et al., 2022; Schar et al., 2020; Vincent et al., 2019; Watts et al., 2017). It is clear that reduction of excessive antimicrobial use in salmon aquaculture will require robust dialogue among all stakeholders (industry, government, workers and communities, and the scientific community). Failure to do this will not only damage piscine, human and environmental health, but also has the potential to result in collapse of an important industry with consequent major economic and societal impacts (Cabello et al., 2013; Flores‐Kossack et al., 2020; Lin, 1989).

## AUTHOR CONTRIBUTIONS

**Felipe C. Cabello:** Conceptualization (equal); validation (equal); writing – original draft (equal); writing – review and editing (equal). **Ana R. Millanao:** Conceptualization (equal); validation (equal); writing – original draft (equal); writing – review and editing (equal). **Ivonne Lozano‐Muñoz:** Conceptualization (equal); validation (equal); writing – original draft (equal); writing – review and editing (equal). **Henry P. Godfrey:** Conceptualization (equal); validation (equal); writing – original draft (equal); writing – review and editing (equal).

## CONFLICT OF INTEREST STATEMENT

The authors declare no conflict of interest.

## Data Availability

All the data discussed in this Opinion is available in the references.
